# Association pathways between AI training recommendation dependence and athletes’ psychological characteristics related to autonomous judgment

**DOI:** 10.3389/fpsyg.2026.1918082

**Published:** 2026-10-01

**Authors:** Xiaogan Chen

**Affiliations:** Department of Physical Education, Taihu University of Wuxi, Wuxi, China

**Keywords:** AI training recommendation dependence, body signal outsourcing, sense of self-regulation, training decision hesitation, training self-efficacy

## Abstract

**Introduction:**

While AI-generated training recommendations may enhance the scientific rigor of competitive sports training, their use may also be associated with athletes’ autonomy in interpreting bodily states and making training decisions. Drawing on cognitive offloading theory, self-determination theory, and the appropriate reliance framework, this study examined a serial association model linking AI training recommendation dependence, body signal outsourcing tendency, reduced sense of self-regulation, training decision hesitation, and reduced training self-efficacy.

**Methods:**

Cross-sectional questionnaire data were collected from 381 athletes. The hypothesized relationships were tested using structural equation modeling, bootstrap analyses of indirect associations, and multigroup analyses comparing athletes in physical-dominant and skill-dominant sports.

**Results:**

AI training recommendation dependence was significantly and positively associated with body signal outsourcing tendency, which was, in turn, positively associated with a reduced sense of self-regulation. A reduced sense of self-regulation was positively associated with training decision hesitation, which was further positively associated with reduced training self-efficacy. Bootstrap analyses identified a significant serial indirect association between AI training recommendation dependence and reduced training self-efficacy through body signal outsourcing tendency, reduced sense of self-regulation, and training decision hesitation. Multigroup analyses further indicated that the positive association between AI training recommendation dependence and body signal outsourcing tendency was stronger among athletes in physical-dominant sports than among those in skill-dominant sports.

**Discussion:**

The findings reveal a continuous pattern of statistical associations linking AI training recommendation dependence with body signal outsourcing tendency, reduced sense of self-regulation, training decision hesitation, and reduced training self-efficacy. These findings highlight the importance of maintaining athletes’ bodily awareness and capacity for autonomous decision-making when integrating AI-generated recommendations into competitive sports training.

## Introduction

As competitive sports training systems increasingly shift from experience-based practice toward data-driven and AI-assisted approaches, artificial intelligence has become progressively integrated into athletes’ training processes. In recent years, training recommendation systems supported by wearable technologies, computer vision, and machine learning models have been widely applied to athletic performance analysis, training load management, and athlete health monitoring (Mateus et al., 2025; Reis et al., 2024). By rapidly integrating multiple sources of information and providing real-time feedback, these systems can support more evidence-informed training adjustments and enhance the scientific rigor of training practice. However, when AI-generated recommendations become an important basis for training-related judgments, their use may be associated not only with greater decision-making efficiency but also with how athletes perceive bodily states, interpret training load, and make training decisions.

From the perspective of training psychology, athletic training involves an ongoing process of self-regulation in which athletes continuously perceive bodily states, evaluate training demands, and adjust behavioral strategies. Bodily signals such as fatigue, pain, arousal, movement stability, and recovery status provide important information for judging training tolerance and regulating subsequent behavior. AI-generated training recommendations can supplement these judgments with data-based information. Nevertheless, greater reliance on system-generated prompts may be associated with a lower degree of active integration of athletes’ own bodily sensations and accumulated training experience.

Cognitive offloading theory proposes that individuals use external tools to reduce cognitive demands by transferring part of the burden of memory, judgment, monitoring, or problem-solving to external systems (Risko and Gilbert, 2016). Research on human–AI decision-making similarly suggests that greater reliance on AI recommendations may be associated with lower levels of active analysis and independent judgment, together with a stronger tendency to passively accept algorithmic advice (Schemmer et al., 2023; Gerlich, 2025). Accordingly, this study defines AI training recommendation dependence as an athlete’s psychological tendency to consistently prioritize, await, or rely on AI-generated recommendations when making training-related judgments, while placing relatively less emphasis on bodily sensations, experiential knowledge, and situational interpretation.

From a theoretical perspective, AI training recommendation dependence may be associated with body signal outsourcing tendency, whereby athletes rely more heavily on AI systems to interpret fatigue, pain, recovery, and movement-related states. Body signal outsourcing tendency may, in turn, be associated with a reduced sense of self-regulation. A reduced sense of self-regulation may co-occur with greater training decision hesitation, reflected in behaviors such as waiting for confirmation, repeatedly reconsidering available information, or hesitating to adjust training independently when AI recommendations are unavailable, ambiguous, or inconsistent with athletes’ own sensations. Training decision hesitation may also be associated with lower confidence in one’s ability to evaluate training conditions independently and may coexist with the perception that training judgments are difficult to make without AI-generated guidance, together with reduced training self-efficacy.

Existing research on AI in competitive sports training has primarily focused on training load prediction, movement recognition, injury risk identification, performance analysis, and the generation of individualized training plans. Much of this literature has emphasized the value of AI for improving monitoring accuracy, supporting training decisions, and enhancing athletic performance (Reis et al., 2024; Musat et al., 2024; Pietraszewski et al., 2025). By contrast, considerably less attention has been paid to the psychological dependence that may accompany sustained exposure to AI-generated training recommendations or to the psychological characteristics associated with bodily awareness and autonomous judgment.

Against this background, the present study develops a serial association model linking AI training recommendation dependence, body signal outsourcing tendency, reduced sense of self-regulation, training decision hesitation, and reduced training self-efficacy. The study examines the continuous statistical associations among these variables and further investigates whether the relevant pathways differ across sport types. In doing so, it extends research on AI-assisted training beyond questions of technical efficacy to encompass athletes’ psychological adaptation to intelligent training environments. The study thereby provides a theoretical and empirical basis for understanding the relationship between AI training recommendation dependence and psychological characteristics related to athletes’ autonomous judgment, while also offering practical guidance for the appropriate use of AI training recommendation systems and the preservation of athletes’ self-regulatory capacities.

## Theoretical foundations and research hypotheses

### Theoretical foundations

#### The cognitive offloading logic between reliance on AI training recommendations and bodily-signal outsourcing

Cognitive offloading theory proposes that individuals use external tools to reduce cognitive demands by transferring portions of memory, judgment, monitoring, or problem-solving tasks to the external environment or technological systems (Risko and Gilbert, 2016). Moderate cognitive offloading is generally associated with greater task-processing efficiency. However, when external tools assume a larger share of perceptual and judgment-related functions that would otherwise require active individual engagement, individuals may rely less on their own experience and judgment.

In AI-assisted training contexts, training recommendation systems can continuously evaluate athletes’ fatigue, recovery status, movement-related risks, and training load using data derived from wearable devices, movement recognition technologies, and algorithmic models. Such feedback can provide data-driven support for training-related judgments and may also be associated with how athletes process bodily information. In conventional training contexts, athletes typically interpret their physical condition by integrating sensations of fatigue and pain, movement stability, and accumulated training experience. By contrast, when AI-generated recommendations constitute a prominent source of training information, athletes may be more inclined to await system prompts and prioritize algorithmic outputs when interpreting their bodily states.

Accordingly, AI training recommendation dependence reflects more than trust in technological information; it also represents greater reliance on external systems when making training-related judgments. When athletes consistently prioritize AI feedback, await system confirmation, or use algorithmic outputs to evaluate their own bodily sensations, their active recognition of fatigue, pain, recovery, and movement-related states may remain at a relatively low level. Bodily signals ordinarily require integrated interpretation based on subjective sensations, training experience, and situational judgment. At higher levels of AI training recommendation dependence, however, the interpretation of such signals may rely more heavily on external systems and may be associated with a stronger body signal outsourcing tendency.

#### Autonomy-related association between externalized bodily perception and reduced sense of self-regulation (self-determination theory)

Self-determination theory posits that the quality of individual behavior depends not only on external demands or behavioral intensity but also on the extent to which individuals experience autonomy, competence, and internal endorsement during action. Among these psychological needs, autonomy refers to the experience of acting in accordance with one’s own volition and internal judgment and constitutes an important foundation for sustained engagement and adaptive development (Deci and Ryan, 2000; Ryan and Deci, 2017).

In training contexts, the recognition and interpretation of bodily signals represent an important basis for athletes’ self-regulation. Signals such as fatigue, pain, recovery status, and movement stability provide direct information for evaluating training tolerance and adjusting training behavior. When athletes actively perceive and interpret these signals, their training regulation is more likely to be grounded in self-judgment and accompanied by a stronger sense of control over the training process. By contrast, greater reliance on AI systems may be associated with more frequent external confirmation and relatively less reliance on internal judgment.

From an autonomy perspective, body signal outsourcing may be associated with a relatively lower sense of personal agency in training-related behavior. Rather than primarily relying on their own bodily sensations and training experience to determine whether an adjustment is needed, athletes may be more inclined to await AI-based confirmation regarding whether and how training should be modified. This externalization of the basis for judgment may be accompanied by greater passivity in training regulation. Even when athletes continue to complete prescribed training tasks, their regulatory behavior may depend more heavily on external prompts and algorithmic confirmation than on their own active interpretation of bodily states and training goals.

#### Human–AI reliance association between imbalanced AI recommendation adoption and training decision hesitation (appropriate reliance framework in human–AI decision making)

The appropriate reliance framework explains how individuals balance algorithmic recommendations with their own judgment when using AI-assisted decision support. According to this framework, effective human–AI decision-making requires individuals to rely on AI recommendations when they are sufficiently reliable while retaining the capacity for independent judgment when those recommendations are uncertain or conflict with their own assessments (Schemmer et al., 2023). Accordingly, effective human–AI collaboration depends not only on the availability of AI-generated recommendations but also on users’ ability to evaluate their appropriateness within a given context.

In AI-assisted training contexts, athletes typically encounter a continuous stream of recommendations concerning fatigue, recovery, movement-related risks, and training load adjustments rather than isolated or unequivocal technical instructions. Because these recommendations are derived from data-driven analyses, athletes may perceive them as relatively objective and authoritative sources of information. When athletes report a relatively reduced sense of self-regulation, confidence in their own bodily sensations and training experience may also be comparatively low. This pattern may be associated with a weaker capacity to balance AI-generated recommendations against personal judgment. A reduced capacity to evaluate the appropriateness of AI recommendations may, in turn, coexist with a greater imbalance in recommendation adoption.

From a human–AI reliance perspective, imbalanced recommendation adoption may be associated with greater training decision hesitation. When AI recommendations are unavailable, ambiguous, or inconsistent with athletes’ own bodily sensations, athletes may be less inclined to rely immediately on their experiential judgment. Instead, they may await system confirmation, repeatedly consult available data, seek additional external reassurance, or hesitate to adjust their training independently. Thus, although AI recommendations do not directly replace athletes’ training decisions, greater reliance on external confirmation may coexist with more pronounced training decision hesitation.

### Research hypotheses

Drawing on cognitive offloading theory, self-determination theory, and the appropriate reliance framework, the present study identifies bodily-signal outsourcing tendency, reduced self-regulatory experience, and training decision hesitancy as key psychological mechanisms linking dependence on AI training recommendations to diminished training self-efficacy. Accordingly, a sequential model is proposed: dependence on AI training recommendations → bodily-signal outsourcing tendency → reduced self-regulatory experience → training decision hesitancy → diminished training self-efficacy. The study further examines whether the proposed associations vary across sport types.

#### H1: dependence on AI training recommendations and bodily-signal outsourcing tendency

Cognitive offloading theory posits that individuals use external tools to reduce cognitive demands associated with judgment and monitoring. In AI-assisted training contexts, training recommendation systems can continuously provide information regarding fatigue, recovery status, movement-related risks, and training load adjustments. When athletes consistently prioritize AI-generated recommendations when evaluating their bodily states, lower levels of active bodily awareness and reduced reliance on experiential judgment may coexist with a stronger body signal outsourcing tendency.

*H1a*: Greater dependence on AI training recommendations is positively associated with a stronger bodily-signal outsourcing tendency.

#### H2: bodily-signal outsourcing tendency and reduced self-regulatory experience

Self-determination theory emphasizes that autonomy is grounded in individuals’ experience of volition, personal agency, and self-directed regulation. In athletic training, effective self-regulation requires athletes to integrate external feedback with bodily sensations, prior experience, and training goals. When the interpretation of physical condition becomes increasingly dependent on AI-generated information, the basis of training regulation may shift from internally generated judgment to external algorithmic confirmation. Such a shift may be associated with a diminished sense of personal control over the training process and, consequently, reduced self-regulatory experience.

*H2a*: A stronger bodily-signal outsourcing tendency is positively associated with reduced self-regulatory experience.

*H2b*: Dependence on AI training recommendations is indirectly associated with reduced self-regulatory experience through bodily-signal outsourcing tendency.

#### H3: reduced self-regulatory experience and training decision hesitancy

Training decisions require athletes to make timely judgments about whether to adjust training load, modify movement execution, alter training intensity, or report physical discomfort. When self-regulatory experience is reduced, athletes may have less confidence in their bodily sensations and accumulated training experience as valid bases for judgment. Under such conditions, they may become more likely to wait for external confirmation, repeatedly reconsider available information, or hesitate to make independent training adjustments. Reduced self-regulatory experience may therefore be associated with greater training decision hesitancy.

*H3a*: Greater reduction in self-regulatory experience is positively associated with greater training decision hesitancy.

*H3b*: Bodily-signal outsourcing tendency is indirectly associated with training decision hesitancy through reduced self-regulatory experience.

#### H4: training decision hesitancy and diminished training self-efficacy

Training self-efficacy reflects athletes’ confidence in their ability to accurately assess their training status, regulate training behavior, and effectively perform training-related tasks. Greater training decision hesitancy is likely to coincide with more frequent uncertainty regarding one’s own judgments and greater reliance on external confirmation. Repeated hesitation may therefore be associated with less favorable evaluations of one’s capacity to regulate training independently and, consequently, with diminished training self-efficacy.

*H4a*: Greater training decision hesitancy is positively associated with diminished training self-efficacy.

*H4b*: Reduced self-regulatory experience is indirectly associated with diminished training self-efficacy through training decision hesitancy.

#### H5: sequential indirect association between dependence on AI training recommendations and diminished training self-efficacy

Taken together, the theoretical relationships outlined above suggest a sequential pattern of associations among dependence on AI training recommendations, bodily-signal outsourcing tendency, reduced self-regulatory experience, training decision hesitancy, and diminished training self-efficacy. Greater dependence on AI-generated recommendations may be associated with a stronger tendency to outsource the interpretation of bodily signals. This tendency may, in turn, correspond with reduced self-regulatory experience, which may be associated with greater hesitation in training-related decisions and, ultimately, diminished training self-efficacy.

*H5a*: Dependence on AI training recommendations is sequentially and indirectly associated with diminished training self-efficacy through bodily-signal outsourcing tendency, reduced self-regulatory experience, and training decision hesitancy.

#### H6: the moderating role of sport type

Different sport types vary in their training load structures, physiological feedback demands, and reliance on data monitoring. Compared with skill-dominant sports, physical-dominant sports typically place greater emphasis on training load regulation, fatigue monitoring, and recovery assessment, resulting in more frequent exposure to data-driven feedback. Accordingly, the positive association between AI training recommendation dependence and body signal outsourcing tendency may be stronger among athletes in physical-dominant sports. The strength of this association may therefore vary across sport types, reflecting differences in training characteristics and patterns of technology use.

*H6a*: Sport type moderates the association between dependence on AI training recommendations and bodily-signal outsourcing tendency, such that this association is stronger in physically demanding sports than in skill-dominant sports.

In summary, the hypothesized model examined in the present study is presented in Figure 1.

**Figure 1:**
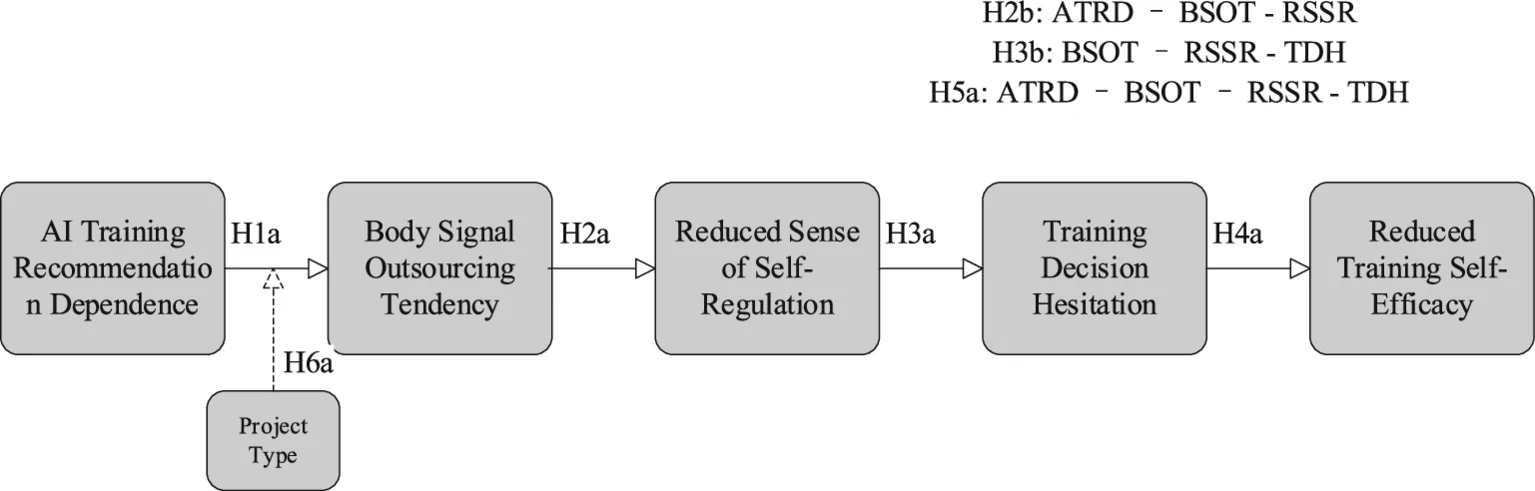
Conceptual model of the study. ATRD, AI Training Recommendation Dependence; BSOT, Body Signal Outsourcing Tendency; RSSR, Reduced Sense of Self-Regulation; TDH, Training Decision Hesitation; RTSE, Reduced Training Self-Efficacy; PT, Project Type.

## Research design

### Study population and sample sources

This study employed non-probability sampling and recruited athletes currently engaged in competitive training within China’s competitive sports training system. Participants were primarily drawn from high-level university sports teams, provincial and municipal professional sports teams, and sports training centers. In this study, an “AI training recommendation system” was defined as a system that uses algorithmic models to analyze athletes’ training, physical condition, or performance data and subsequently generates recommendations concerning training load adjustments, movement corrections, recovery scheduling, risk warnings, or personalized training. Devices or platforms that merely provide raw monitoring data, display performance metrics, or deliver routine data feedback without generating training recommendations were not classified as AI training recommendation systems.

To ensure alignment between the sample and the research focus, participants were required to meet all of the following criteria: (1) be engaged in regular competitive training at the time of the survey; (2) have practical experience using the AI training recommendation systems defined above; and (3) have actually received system-generated recommendations concerning training load adjustment, movement correction, recovery assessment, risk warning, or personalized training during routine training and be able to complete the questionnaire based on their own usage experience. This population was selected because its members are regularly exposed to AI-generated training recommendations, making it suitable for examining the patterns of association among AI training recommendation dependence, body signal outsourcing tendency, reduced sense of self-regulation, and training decision hesitation.

The sample was recruited from multiple regions of China, including East, North, Central, South, and Southwest China, and covered Jiangsu, Shandong, Beijing, Hubei, Hunan, Guangdong, Sichuan, Fujian, and Zhejiang. Restricting the sample to Chinese athletes helped reduce potential cross-cultural heterogeneity in the relationships among the study variables and allowed the proposed model to be examined within a relatively consistent context of training systems, coaching management, and technology use. Participants represented a broad range of sports, including track and field, swimming, rowing, cycling, basketball, volleyball, soccer, wushu, gymnastics, table tennis, and badminton. This diversity also provided an appropriate basis for examining sport type as a potential moderator.

The individual athlete served as the unit of analysis. A total of 489 questionnaires were distributed, of which 432 were returned. To enhance transparency and data quality, questionnaires were excluded if they exhibited obvious response-pattern uniformity, substantial missing data, implausible completion times, or if respondents had not actually been exposed to AI training recommendations or data-driven training feedback. Based on these criteria, 51 questionnaires were excluded, yielding a final valid sample of 381 and a valid response rate of 77.9%. Before the formal survey, a pilot test was conducted with 25 athletes; these participants were not included in the final analysis. Feedback from the pilot test was used to revise items that were unclear or potentially ambiguous and to standardize questionnaire instructions, administration procedures, and data-screening criteria.

The study received ethical approval from the Academic Ethics Review Committee of Wuxi Taihu University before formal data collection (Approval No. HREC-2026-033). All participants took part voluntarily after providing informed consent. Participants under 18 years of age were included only after written informed consent had been obtained from their legal guardians and assent had been obtained from the participants themselves. All questionnaires were completed anonymously, and the collected data were used exclusively for academic research. No personally identifiable information was included in the reporting of the results. The study procedures were conducted in accordance with the Declaration of Helsinki and relevant institutional ethical standards.

The final sample comprised athletes aged 16–25 years, with a mean age of 19.6 years (SD = 2.2). Of the 381 participants, 208 were male (54.6%) and 173 were female (45.4%). By sport type, 196 athletes (51.4%) participated in physically dominant sports, whereas 185 (48.6%) participated in skill-dominant sports. The physically dominant group comprised 45 track-and-field athletes, 51 swimmers, 44 rowers, and 56 cyclists. The skill-dominant group included 35 gymnasts, 24 wushu athletes, 28 table tennis players, 26 badminton players, 27 basketball players, 30 volleyball players, and 15 soccer players. With respect to athletic ranking, 86 participants (22.6%) held National Class 1 status or above, 171 (44.9%) held National Class 2 status, and 124 (32.5%) had no official athletic ranking. Participants had trained for an average of 7.0 years (SD = 2.7), and most reported training between five and nine sessions per week. Regarding experience with AI-assisted training technologies, 89 athletes (23.4%) had used intelligent training devices or AI-based recommendation systems for less than 6 months, 135 (35.4%) for 6 months to 1 year, and 157 (41.2%) for more than 1 year.

### Measurement instruments

All core variables were assessed using a 5-point Likert scale ranging from 1 (“strongly disagree”) to 5 (“strongly agree”). Scores for each variable were calculated by averaging the corresponding item scores, with higher scores indicating higher levels of the respective construct. In this study, “AI training recommendations” specifically refer to training assessments or actionable recommendations generated by an AI training recommendation system through algorithmic analysis of athletes’ training, physical condition, or performance data. Devices or platforms that merely provide raw monitoring data, display performance metrics, or deliver routine data feedback without generating training recommendations were not included within this definition.

Because AI training recommendation dependence, body signal outsourcing tendency, reduced sense of self-regulation, and training decision hesitation are highly context-specific constructs associated with intelligent training environments, existing scales were not fully suited to the specific research questions addressed in this study. Accordingly, items were developed or adapted on the basis of relevant theories and previous research to reflect the competitive training context. The questionnaire was administered in Chinese. For context-specific items informed by English-language theories and literature, the Chinese wording was formulated to preserve the theoretical meaning of the original constructs while ensuring contextual appropriateness for the Chinese competitive sports training context.

To enhance the conceptual distinctiveness of closely related constructs, the item-development process differentiated the measurement focus of each construct according to its theoretical definition. AI training recommendation dependence captures athletes’ reliance on AI as an external source of training advice; body signal outsourcing tendency reflects the extent to which the interpretation of bodily states is delegated to external systems; reduced sense of self-regulation captures lower levels of perceived autonomy and control over training regulation; training decision hesitation reflects hesitation, waiting, and repeated confirmation in specific training decision-making situations; and reduced training self-efficacy captures lower confidence in athletes’ ability to independently evaluate and regulate their training.

Before the formal survey, three experts in sport psychology and sports training reviewed the items for relevance, clarity, and contextual appropriateness. Item relevance was evaluated primarily in terms of the correspondence between item content and the predefined construct definitions, thereby helping to ensure adequate representation of the intended theoretical domains. A pilot test was subsequently conducted with 25 athletes. Based on their feedback, minor wording revisions were made, particularly to items identified as difficult to understand or potentially ambiguous.

Dependence on AI training recommendations was assessed as the extent to which athletes prioritized, awaited, or relied on AI-generated recommendations when making training-related decisions. Conceptually, this construct captured athletes’ general reliance on AI as an external source of advice and was not restricted to the interpretation of bodily states such as fatigue, pain, recovery, or movement condition. It was therefore distinct from bodily-signal outsourcing tendency. Item development was primarily informed by cognitive offloading theory and research on reliance on AI recommendations in human–AI decision-making (Risko and Gilbert, 2016; Schemmer et al., 2023). Six items were developed for the AI-assisted training context. Example items included “When deciding whether to adjust my training, I usually prioritize the AI system’s recommendations” and “Without AI training recommendations, I would find it difficult to accurately assess my training status.” Confirmatory factor analysis indicated that all standardized factor loadings were significant and exceeded 0.60 (*p* < 0.001). The scale demonstrated satisfactory internal consistency and convergent validity (Cronbach’s *α* = 0.89, CR = 0.89, AVE = 0.58).

Bodily-signal outsourcing tendency assessed the extent to which athletes delegated the interpretation of bodily signals—including fatigue, pain, recovery status, and movement condition—to external AI systems. This construct focused specifically on how bodily information was interpreted and whether the interpretive task was transferred to an external system. It was therefore distinct from the broader reliance on external advice reflected in dependence on AI training recommendations and from the subjective experience of autonomy and control captured by reduced self-regulatory experience. The scale was developed from the concept of judgment-task offloading within cognitive offloading theory and contextualized to reflect athletes’ experiences with data-driven training feedback (Risko and Gilbert, 2016; Gerlich, 2025). The scale comprised five items. Example items included “I prefer to let the AI system determine whether my body is fatigued” and “I trust the system’s assessment of my recovery status more than my own bodily sensations.” All standardized factor loadings exceeded 0.60 and were statistically significant (*p* < 0.001). The scale demonstrated satisfactory internal consistency and convergent validity (Cronbach’s *α* = 0.88, CR = 0.88, AVE = 0.59).

Reduced self-regulatory experience assessed the extent to which athletes experienced a diminished subjective sense of control over the evaluation of their physical condition, the adjustment of training, and the regulation of training behavior. This construct captured perceived reductions in autonomy and control during training regulation rather than hesitation or waiting behavior in specific decision-making situations and was therefore conceptually distinct from training decision hesitancy. Item development was informed by the conceptualization of autonomy within self-determination theory (Deci and Ryan, 2000; Ryan and Deci, 2017) and adapted to the context of AI-assisted training regulation. The scale comprised five items. Example items included “When it comes to training adjustments, I am increasingly unsure whether my own judgments are reliable” and “Without prompts from an external system, I find it difficult to proactively decide how to adjust my training.” Higher scores indicated greater reductions in self-regulatory experience. Confirmatory factor analysis indicated that all factor loadings met acceptable criteria. The scale demonstrated satisfactory internal consistency and convergent validity (Cronbach’s *α* = 0.87, CR = 0.87, AVE = 0.57).

Training decision hesitancy assessed athletes’ tendencies to hesitate, delay decisions, wait for confirmation, or repeatedly verify information when making decisions concerning training-load adjustment, movement correction, physical discomfort, and training pace. This construct reflected uncertainty and delayed action within specific training decision-making processes and was conceptually distinct from athletes’ broader evaluations of their own training-regulation competence. Item development drew on research concerning miscalibrated reliance, overreliance, and decision uncertainty within the appropriate reliance framework and the broader literature on human–AI collaborative decision-making (Schemmer et al., 2023; Schoeffer et al., 2025). The scale comprised five items. Example items included “When AI recommendations are unclear, I often do not know whether I should adjust my training” and “Even when I feel physically unwell, I wait for further confirmation from the system or data before deciding whether to change my training.” The scale demonstrated satisfactory internal consistency and convergent validity (Cronbach’s *α* = 0.88, CR = 0.88, AVE = 0.60).

Diminished training self-efficacy assessed the extent to which athletes reported reduced confidence in their ability to evaluate their training status, regulate training behavior, and effectively perform training-related tasks. Unlike training decision hesitancy, which referred to uncertainty within specific decision-making processes, this construct focused on a broader decline in athletes’ confidence in their ability to independently evaluate and regulate training. Item development was informed by the core principles of self-efficacy theory (Bandura, 1997) and contextualized to situations involving reliance on AI-generated training recommendations. The scale comprised six items. Example items included “Without AI training recommendations, I lack confidence in my ability to plan my training appropriately” and “I am increasingly doubtful of my ability to independently assess my training status.” Higher scores indicated a greater decline in training self-efficacy. Confirmatory factor analysis showed that all standardized factor loadings were significant and exceeded 0.60 (*p* < 0.001). The scale demonstrated satisfactory internal consistency and convergent validity (Cronbach’s *α* = 0.89, CR = 0.89, AVE = 0.59).

Sport type was self-reported by participants according to their primary discipline and was operationally classified on the basis of training-task structure, dominant performance demands, and the nature of training-status feedback. Drawing on established perspectives in sports training classification (Bompa and Haff, 2009), sports were categorized as physically dominant or skill-dominant according to the relative importance of physical-load information versus technical and situational information in training decision-making. In physically dominant sports, training-load adjustment and condition assessment relied heavily on indicators related to speed, endurance, power, fatigue, recovery, and other quantifiable physiological or performance-related information. This category included track and field, swimming, rowing, and cycling, comprising 196 participants. In skill-dominant sports, training decisions depended not only on physical load but also substantially on technical execution, motor coordination, perceptual judgment, and context-specific information. This category included gymnastics, wushu, table tennis, badminton, basketball, volleyball, and soccer, comprising 185 participants. This classification was intended to capture contextual differences in the application of AI-generated training recommendations across different training-task structures and feedback environments rather than to imply that sports within either category depended exclusively on physical or technical factors. Sport type was subsequently included as the grouping variable in the multi-group analyses.

Age, gender, athletic level, years of training, weekly training frequency, and duration of AI-assisted training-system use were included as control variables to reduce the potential influence of demographic characteristics and differences in training exposure on the core structural relationships. Overall, Cronbach’s α coefficients for all scales exceeded 0.80, CR values exceeded 0.85, and AVE values exceeded 0.50. These results indicated satisfactory internal consistency and convergent validity across the measurement instruments and provided an adequate measurement basis for subsequent confirmatory factor analysis and structural path analysis.

### Data analysis methods

This study used SPSS 26.0 and Mplus 8.3 for data processing and statistical analyses. Prior to the main analyses, the raw data were screened for missing values, outliers, invariant response patterns, implausible response times, and logically inconsistent responses. Questionnaires that failed to meet the predefined data-quality criteria were excluded, resulting in a final analytic sample of 381 athletes. Descriptive statistics, including means, standard deviations, skewness, and kurtosis, were then calculated for all core variables, together with Pearson correlation coefficients to characterize their distributions and bivariate associations.

To assess the potential influence of common method bias, Harman’s single-factor test was conducted, and the fit of a one-factor confirmatory factor model was further compared with that of the hypothesized five-factor model. Harman’s single-factor test was used to determine whether the first factor extracted from the unrotated factor solution accounted for a disproportionately large proportion of the total variance. The one-factor confirmatory factor model was used to examine whether all measurement items could be adequately represented by a single latent factor. These procedures were treated as diagnostic assessments of potential common method variance rather than as definitive tests capable of ruling out common method bias (Podsakoff et al., 2003).

The measurement model was evaluated using confirmatory factor analysis. A five-factor model comprising AI training recommendation dependence, body signal outsourcing tendency, reduced sense of self-regulation, training decision hesitation, and reduced training self-efficacy was specified and compared with a one-factor model and alternative competing models. Model fit was evaluated using *χ*^2^, df, *p*, *χ*^2^/df, CFI, TLI, RMSEA with its 90% confidence interval, and SRMR. Convergent validity was assessed using standardized factor loadings, composite reliability (CR), and average variance extracted (AVE), whereas discriminant validity was evaluated using the Fornell–Larcker criterion (Fornell and Larcker, 1981; Kline, 2016). To provide a more stringent assessment of discriminant validity among the five theoretically related constructs, the heterotrait–monotrait ratio (HTMT) was also examined, with values below 0.85 taken as evidence of adequate discriminant validity. In addition, 5,000 bootstrap resamples were used to estimate 95% confidence intervals for the HTMT values, thereby providing a more robust assessment of construct distinctiveness.

After the measurement model demonstrated acceptable psychometric properties, a recursive path analysis was conducted using the mean scores of the items representing each construct to test the proposed serial association model: “AI Training Recommendation Dependence → Body Signal Outsourcing Tendency → Reduced Sense of Self-Regulation → Training Decision Hesitation → Reduced Training Self-Efficacy.” Age, gender, athletic level, years of training, weekly training frequency, and duration of AI training recommendation system use were included as control variables.

To comprehensively estimate the associations between antecedent variables and subsequent endogenous variables, each endogenous construct was regressed on all preceding core variables and the control variables. Specifically, body signal outsourcing tendency was regressed on AI training recommendation dependence and the control variables; reduced sense of self-regulation was regressed on AI training recommendation dependence, body signal outsourcing tendency, and the control variables; training decision hesitation was regressed on AI training recommendation dependence, body signal outsourcing tendency, reduced sense of self-regulation, and the control variables; and reduced training self-efficacy was regressed on AI training recommendation dependence, body signal outsourcing tendency, reduced sense of self-regulation, training decision hesitation, and the control variables. Thus, the model estimated not only the adjacent paths specified in the theoretical framework but also all direct paths from preceding core variables to subsequent endogenous variables, together with the associations between the control variables and each endogenous construct. This specification constituted a fully recursive saturated path model.

Because the path model was saturated, global fit indices were not informative for assessing model fit. Accordingly, *χ*^2^, CFI, TLI, RMSEA, and SRMR were not interpreted for this model. Instead, emphasis was placed on path coefficients, the explained variance of the endogenous variables, and indirect effects. Model parameters were estimated using maximum likelihood estimation with robust standard errors to account for potential deviations from distributional assumptions. For all paths involving the core and control variables, unstandardized coefficients (B), standard errors (SE), standardized coefficients (β), *z*-values, *p*-values, and 95% confidence intervals were reported to enhance the completeness and reproducibility of the analysis. Serial indirect effects were examined using a bias-corrected nonparametric bootstrap procedure with 5,000 resamples. An indirect effect was considered statistically significant when its corresponding 95% confidence interval did not include zero (Hayes, 2018). Indirect-effect results were reported as standardized indirect effects, bootstrap standard errors, and 95% confidence intervals.

Differences across sport types were examined using a two-stage multigroup analysis. First, multigroup confirmatory factor analysis was conducted to assess measurement invariance between the physical-dominant and skill-dominant sport groups. Configural, metric, and scalar invariance were tested sequentially. The configural invariance model examined whether the two groups shared the same underlying factor structure. The metric invariance model additionally constrained corresponding factor loadings to equality across groups, whereas the scalar invariance model further constrained item intercepts to equality across groups. Measurement invariance was evaluated primarily on the basis of chi-square difference tests and changes in CFI and RMSEA, with |ΔCFI| ≤ 0.010 and |ΔRMSEA| ≤ 0.015 used as criteria supporting invariance.

After measurement invariance had been established, multigroup path analysis based on construct mean scores was conducted to compare the “AI training recommendation dependence → body signal outsourcing tendency” path across the two sport groups. Specifically, this path was constrained to equality across groups and compared with a model in which it was freely estimated. The resulting model comparison was used to determine whether the path coefficient differed significantly between the physical-dominant and skill-dominant sport groups. All statistical tests were two-tailed, with the significance level set at *α* = 0.05.

## Research findings

### Descriptive statistics and correlation analysis

To provide a preliminary assessment of the distributional characteristics, measurement quality, and interrelationships among the core constructs, descriptive statistics, reliability analyses, convergent validity assessments, and Pearson correlation analyses were conducted for dependence on AI training recommendations, bodily-signal outsourcing tendency, reduced self-regulatory experience, training decision hesitancy, and diminished training self-efficacy. The results are presented in Table 1.

**Table 1:** Descriptive statistics, reliability, validity, and correlations.

Variable	*M*	SD	Cronbach’s *α*	CR	AVE	1	2	3	4	5
ATRD	3.18	0.71	0.89	0.89	0.58	**0.76**				
BSOT	3.11	0.73	0.88	0.88	0.59	0.57***	**0.77**			
RSSR	3.04	0.70	0.87	0.87	0.57	0.43***	0.51***	**0.76**		
TDH	2.97	0.72	0.88	0.88	0.60	0.22***	0.34***	0.55***	**0.78**	
RTSE	2.93	0.73	0.89	0.89	0.59	0.22***	0.27***	0.41***	0.54***	**0.77**

*N* = 381. ATRD, AI Training Recommendation Dependence; BSOT, Body Signal Outsourcing Tendency; RSSR, Reduced Sense of Self-Regulation; TDH, Training Decision Hesitation; RTSE, Reduced Training Self-Efficacy. The bold values on the diagonal represent the square roots of AVE. ***p < 0.001.

As shown in Table 1, AI training recommendation dependence (*M* = 3.18, SD = 0.71), body signal outsourcing tendency (*M* = 3.11, SD = 0.73), reduced sense of self-regulation (*M* = 3.04, SD = 0.70), training decision hesitation (*M* = 2.97, SD = 0.72), and reduced training self-efficacy (*M* = 2.93, SD = 0.73) generally fell within the moderate range. These results indicate that psychological characteristics related to AI training recommendation dependence and autonomous judgment were generally at moderate levels in the current sample. Cronbach’s α coefficients ranged from 0.87 to 0.89, composite reliability (CR) values ranged from 0.87 to 0.89, and average variance extracted (AVE) values ranged from 0.57 to 0.60, all of which met commonly accepted psychometric criteria. These findings support the internal consistency and convergent validity of the measurement instruments. In addition, the square roots of the AVE values ranged from 0.76 to 0.78 and exceeded the corresponding inter-construct correlations in all cases, providing evidence of satisfactory discriminant validity.

Pearson correlation analyses showed that dependence on AI training recommendations was significantly and positively correlated with bodily-signal outsourcing tendency (*r* = 0.57, *p* < 0.001). Bodily-signal outsourcing tendency was significantly and positively correlated with reduced self-regulatory experience (*r* = 0.51, *p* < 0.001), which, in turn, was significantly and positively correlated with training decision hesitancy (*r* = 0.55, *p* < 0.001). Training decision hesitancy was also significantly and positively correlated with diminished training self-efficacy (*r* = 0.54, *p* < 0.001). In addition, dependence on AI training recommendations was significantly and positively correlated with reduced self-regulatory experience (*r* = 0.43, *p* < 0.001), training decision hesitancy (*r* = 0.22, *p* < 0.001), and diminished training self-efficacy (*r* = 0.22, *p* < 0.001). Overall, the observed correlation pattern was consistent with the proposed theoretical relationships and provided preliminary statistical support for the subsequent path analyses and tests of sequential indirect associations.

### Measurement model validation: confirmatory factor analysis (CFA)

To further evaluate the measurement structure of the five latent constructs—dependence on AI training recommendations, bodily-signal outsourcing tendency, reduced self-regulatory experience, training decision hesitancy, and diminished training self-efficacy—confirmatory factor analysis (CFA) was conducted to test the hypothesized five-factor measurement model. The model demonstrated excellent fit to the data, *χ*^2^(314) = 343.232, *p* = 0.123, *χ*^2^/df = 1.09, CFI = 0.995, TLI = 0.994, RMSEA = 0.016, 90% CI [0.000, 0.026], and SRMR = 0.034. The non-significant *χ*^2^ statistic, high incremental fit indices, low residual-based fit indices, and low upper bound of the RMSEA confidence interval collectively indicated that the hypothesized five-factor structure provided a highly satisfactory representation of the observed data.

At the item level, all standardized factor loadings on their respective latent constructs were statistically significant (*p* < 0.001) and exceeded 0.60, indicating that the observed items adequately represented their intended constructs. Consistent with the results reported in Table 1, Cronbach’s α coefficients ranged from 0.87 to 0.89, CR values ranged from 0.87 to 0.89, and AVE values ranged from 0.57 to 0.60. These values met commonly accepted psychometric criteria and supported satisfactory internal consistency and convergent validity across the five constructs.

To further evaluate the distinctiveness of the proposed measurement structure, the hypothesized five-factor model was compared with four-factor, three-factor, two-factor, and single-factor alternatives, as shown in Table 2. The five-factor model consistently outperformed all competing models. When dependence on AI training recommendations and bodily-signal outsourcing tendency were combined into a single factor, the resulting four-factor model showed substantially poorer fit, *χ*^2^(318) = 756.460, *p* < 0.001, *χ*^2^/df = 2.38, CFI = 0.921, TLI = 0.912, RMSEA = 0.060, 90% CI [0.055, 0.066], and SRMR = 0.058. Model fit deteriorated further as additional constructs were combined in the three-factor and two-factor models. The single-factor model showed the poorest fit, *χ*^2^(324) = 2845.274, *p* < 0.001, *χ*^2^/df = 8.78, CFI = 0.543, TLI = 0.505, RMSEA = 0.143, 90% CI [0.138, 0.148], and SRMR = 0.145. These results indicated that the measurement items could not be adequately represented by a single common construct. More broadly, the progressive deterioration in model fit as conceptually distinct constructs were combined provided additional support for treating the five variables as empirically distinguishable latent constructs.

**Table 2:** Comparison of measurement models.

Model	*χ*^2^/df	CFI	TLI	RMSEA	90% CI for RMSEA	SRMR	*P*
Five-factor model	1.09	0.995	0.994	0.016	[0.000, 0.026]	0.034	0.123
Four-factor model	2.38	0.921	0.912	0.060	[0.055, 0.066]	0.058	< 0.001
Three-factor model	3.70	0.843	0.828	0.084	[0.079, 0.089]	0.082	< 0.001
Two-factor model	5.55	0.733	0.710	0.109	[0.105, 0.114]	0.109	< 0.001
One-factor model	8.78	0.543	0.505	0.143	[0.138, 0.148]	0.145	< 0.001

The five-factor model specified ATRD, BSOT, RSSR, TDH, and RTSE as five distinct latent variables. The four-factor model combined ATRD and BSOT. The three-factor model combined ATRD with BSOT and RSSR with TDH. The two-factor model combined ATRD, BSOT, and RSSR into one factor and TDH and RTSE into another factor. The one-factor model loaded all items onto a single latent factor. ATRD, AI Training Recommendation Dependence; BSOT, Body Signal Outsourcing Tendency; RSSR, Reduced Sense of Self-Regulation; TDH, Training Decision Hesitation; RTSE, Reduced Training Self-Efficacy.

Discriminant validity was further assessed using the heterotrait–monotrait ratio of correlations (HTMT). Across the 381 valid cases, pairwise HTMT values ranged from 0.245 to 0.650, all below the conservative threshold of 0.85. The highest HTMT value was observed between dependence on AI training recommendations and bodily-signal outsourcing tendency (HTMT = 0.650), followed by reduced self-regulatory experience and training decision hesitancy (HTMT = 0.627), and training decision hesitancy and diminished training self-efficacy (HTMT = 0.607). All remaining HTMT values were below 0.60. Bootstrap analyses based on 5,000 resamples further showed that the upper bounds of the 95% confidence intervals for all construct pairs remained below 0.85. Specifically, the 95% confidence interval was [0.582, 0.712] for dependence on AI training recommendations and bodily-signal outsourcing tendency, [0.542, 0.703] for reduced self-regulatory experience and training decision hesitancy, and [0.521, 0.685] for training decision hesitancy and diminished training self-efficacy. These findings provided further evidence that even conceptually adjacent constructs remained empirically distinguishable.

To assess the potential influence of common-method bias, Harman’s single-factor test was conducted using the 27 core measurement items. The unrotated principal component analysis identified five factors with eigenvalues greater than 1, and the first factor accounted for 34.68% of the total variance, below the commonly used 40% warning threshold. Consistent with this result, the confirmatory single-factor model showed poor fit, *χ*^2^(324) = 2845.274, *p* < 0.001, CFI = 0.543, TLI = 0.505, RMSEA = 0.143, and SRMR = 0.145, and performed substantially worse than the hypothesized five-factor model. Taken together, these diagnostic results did not suggest that the observed covariance structure was dominated by a single common factor. Nevertheless, because all core variables were assessed through athletes’ self-reports collected at the same time point, these statistical diagnostics could not completely exclude the possibility of common-method variance. This limitation is therefore addressed further in the discussion of the study limitations.

### Structural path analysis

After the measurement model demonstrated acceptable psychometric properties, a recursive path analysis based on construct mean scores was conducted to examine the associations among AI training recommendation dependence, body signal outsourcing tendency, reduced sense of self-regulation, training decision hesitation, and reduced training self-efficacy. The primary theoretical chain was specified as “AI Training Recommendation Dependence → Body Signal Outsourcing Tendency → Reduced Sense of Self-Regulation → Training Decision Hesitation → Reduced Training Self-Efficacy.” Age, gender, athletic level, years of training, weekly training frequency, and duration of AI training recommendation system use were included as control variables to account for potential background-related variation in the estimated associations. Figure 2 presents only the core theoretical chain, whereas the additional direct paths among the core variables and the paths involving the control variables are reported below. Standardized path coefficients are displayed in Figure 2.

**Figure 2:**
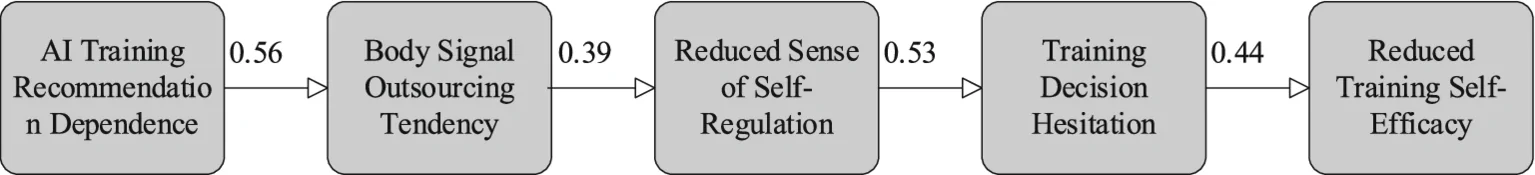
Standardized path model.

AI training recommendation dependence was significantly and positively associated with body signal outsourcing tendency (*β* = 0.56, *p* < 0.001), indicating that higher levels of AI training recommendation dependence were associated with higher levels of body signal outsourcing tendency. This finding supports H1a and provides evidence of a significant association between AI training recommendation dependence and the externalization of bodily-state interpretation.

Body signal outsourcing tendency was significantly and positively associated with reduced sense of self-regulation (*β* = 0.39, *p* < 0.001). Reduced sense of self-regulation was, in turn, significantly and positively associated with training decision hesitation (*β* = 0.53, *p* < 0.001). Thus, athletes reporting a more pronounced reduction in perceived self-regulation also tended to report greater hesitation, repeated confirmation seeking, and reluctance to make independent training adjustments when confronted with changes in training load, physical discomfort, or ambiguous AI recommendations. Training decision hesitation was also significantly and positively associated with reduced training self-efficacy (*β* = 0.44, *p* < 0.001). These findings support H2a, H3a, and H4a, respectively. The corresponding unstandardized coefficients for the four core paths were *B* = 0.580, 0.378, 0.543, and 0.449, with standard errors of 0.044, 0.051, 0.053, and 0.052, respectively. The 95% confidence intervals for all four paths excluded zero, further supporting the statistical significance of these associations.

In addition to the adjacent paths specified in the theoretical model, the fully recursive model estimated all direct paths from preceding core variables to subsequent endogenous variables. AI training recommendation dependence was significantly and positively associated with reduced sense of self-regulation (*B* = 0.202, SE = 0.053, *β* = 0.204, *z* = 3.829, *p* < 0.001, 95% CI [0.098, 0.306]). By contrast, its direct associations with training decision hesitation (*B* = −0.063, SE = 0.055, *β* = −0.062, *z* = −1.148, *p* = 0.252, 95% CI [−0.171, 0.045]) and reduced training self-efficacy (*B* = 0.060, SE = 0.055, *β* = 0.058, z = 1.090, *p* = 0.276, 95% CI [−0.048, 0.168]) were not statistically significant. Body signal outsourcing tendency showed a significant positive direct association with training decision hesitation (*B* = 0.111, SE = 0.056, *β* = 0.113, z = 1.993, *p* = 0.047, 95% CI [0.001, 0.221]), whereas its direct association with reduced training self-efficacy was not significant (*B* = 0.034, SE = 0.056, *β* = 0.034, *z* = 0.609, *p* = 0.543, 95% CI [−0.076, 0.145]). Reduced sense of self-regulation was also significantly and positively associated with reduced training self-efficacy (*B* = 0.145, SE = 0.060, *β* = 0.140, *z* = 2.417, *p* = 0.016, 95% CI [0.027, 0.263]). Overall, the four core associations in the proposed theoretical chain remained statistically significant after the additional direct paths were simultaneously estimated.

The control-variable analyses showed that, in the body signal outsourcing tendency equation, age (*B* = 0.006, *β* = 0.016, *p* = 0.718), gender (*B* = −0.037, *β* = −0.025, *p* = 0.545), athletic level (*B* = 0.049, *β* = 0.049, *p* = 0.242), years of training (*B* = 0.012, *β* = 0.045, *p* = 0.330), and weekly training frequency (*B* = −0.017, *β* = −0.028, *p* = 0.501) were not statistically significant. Duration of AI training recommendation system use showed a weak but significant positive association with body signal outsourcing tendency (*B* = 0.090, SE = 0.040, *β* = 0.096, *z* = 2.251, *p* = 0.025, 95% CI [0.011, 0.168]). In the reduced sense of self-regulation equation, none of the control variables reached statistical significance: age (*B* = 0.007, *β* = 0.020, *p* = 0.664), gender (*B* = −0.041, *β* = −0.029, *p* = 0.497), athletic level (*B* = −0.066, *β* = −0.069, *p* = 0.112), years of training (*B* = 0.018, *β* = 0.071, *p* = 0.132), weekly training frequency (*B* = 0.016, *β* = 0.026, *p* = 0.543), or duration of AI training recommendation system use (*B* = 0.031, *β* = 0.035, *p* = 0.430).

Similarly, in the training decision hesitation equation, age (*B* = −0.002, *β* = −0.005, *p* = 0.919), gender (*B* = 0.048, *β* = 0.033, *p* = 0.439), athletic level (*B* = 0.006, *β* = 0.006, *p* = 0.895), years of training (*B* = 0.001, *β* = 0.003, *p* = 0.958), weekly training frequency (*B* < 0.001, *β* = −0.001, *p* = 0.989), and duration of AI training recommendation system use (*B* = −0.069, *β* = −0.076, *p* = 0.088) were not statistically significant. In the reduced training self-efficacy equation, age showed a weak but significant negative association (*B* = −0.036, SE = 0.017, *β* = −0.100, *z* = −2.169, *p* = 0.031, 95% CI [−0.069, −0.003]), whereas gender (*B* = −0.006, *β* = −0.004, *p* = 0.928), athletic level (*B* = −0.013, *β* = −0.013, *p* = 0.767), years of training (*B* = 0.002, *β* = 0.007, *p* = 0.881), weekly training frequency (*B* = 0.043, *β* = 0.069, *p* = 0.106), and duration of AI training recommendation system use (*B* = −0.047, *β* = −0.050, *p* = 0.252) were not statistically significant.

Overall, apart from the weak association between duration of AI training recommendation system use and body signal outsourcing tendency and the weak association between age and reduced training self-efficacy, none of the remaining control-variable paths reached statistical significance. The core theoretical associations remained statistically significant after adjustment for these background variables.

With respect to explanatory power, dependence on AI training recommendations and the control variables jointly accounted for 34.4% of the variance in bodily-signal outsourcing tendency. Dependence on AI training recommendations, bodily-signal outsourcing tendency, and the control variables jointly accounted for 30.6% of the variance in reduced self-regulatory experience. The antecedent variables accounted for 31.6% of the variance in training decision hesitancy, whereas the final model accounted for 33.2% of the variance in diminished training self-efficacy. Overall, the model demonstrated meaningful explanatory power across the endogenous variables and provided empirical support for the proposed sequential pattern of associations linking dependence on AI-generated training recommendations to psychological characteristics related to athletes’ autonomous judgment.

Sequential indirect associations were further examined using a nonparametric bootstrap procedure with 5,000 resamples. The indirect association between dependence on AI training recommendations and reduced self-regulatory experience through bodily-signal outsourcing tendency was statistically significant (*β* = 0.221, 95% CI [0.156, 0.295]). Similarly, bodily-signal outsourcing tendency was indirectly associated with training decision hesitancy through reduced self-regulatory experience (*β* = 0.209, 95% CI [0.145, 0.281]). Reduced self-regulatory experience was also indirectly associated with diminished training self-efficacy through training decision hesitancy (*β* = 0.235, 95% CI [0.167, 0.306]). The corresponding bootstrap standard errors were 0.035, 0.035, and 0.036, respectively, and none of the 95% confidence intervals included zero. These findings supported H2b, H3b, and H4b, respectively.

The full sequential indirect association between dependence on AI training recommendations and diminished training self-efficacy through bodily-signal outsourcing tendency, reduced self-regulatory experience, and training decision hesitancy was also statistically significant (*β* = 0.052, bootstrap SE = 0.012, 95% CI [0.031, 0.079]). Because the confidence interval excluded zero, H5a was supported. Complete parameter estimates for the core direct associations and bootstrap indirect associations are reported in Table 3.

**Table 3:** Structural path and indirect effect estimates.

Effect type	Path	*B*	SE/Bootstrap SE	*β*	*z*	*p*	95% CI
Direct	ATRD → BSOT	0.580	0.044	0.562	13.131	<0.001	[0.493, 0.666]
Direct	BSOT → RSSR	0.378	0.051	0.393	7.347	<0.001	[0.277, 0.478]
Direct	RSSR → TDH	0.543	0.053	0.531	10.293	<0.001	[0.440, 0.646]
Direct	TDH → RTSE	0.449	0.052	0.443	8.613	<0.001	[0.347, 0.551]
Indirect	ATRD → BSOT → RSSR	–	0.035	0.221	–	–	[0.156, 0.295]
Indirect	BSOT → RSSR → TDH	–	0.035	0.209	–	–	[0.145, 0.281]
Indirect	RSSR → TDH → RTSE	–	0.036	0.235	–	–	[0.167, 0.306]
Indirect	ATRD → BSOT → RSSR → TDH → RTSE	–	0.012	0.052	–	–	[0.031, 0.079]

*N* = 381. B = unstandardized coefficient; SE = standard error; β = standardized coefficient. The path analysis was conducted using construct mean scores. For direct effects, the 95% CIs refer to the unstandardized coefficients. For indirect effects, SE represents the Bootstrap standard error, and the 95% CIs are bias-corrected Bootstrap confidence intervals based on 5,000 resamples.

Taken together, the findings revealed a coherent sequential pattern of statistical associations among dependence on AI training recommendations, bodily-signal outsourcing tendency, reduced self-regulatory experience, training decision hesitancy, and diminished training self-efficacy. Greater dependence on AI-generated training recommendations was associated with stronger bodily-signal outsourcing tendency, which was further associated with reduced self-regulatory experience. Reduced self-regulatory experience was, in turn, associated with greater training decision hesitancy, which corresponded with diminished training self-efficacy. The significant full sequential indirect association provided further empirical support for the proposed model. Nevertheless, because the analysis was based on cross-sectional data, these findings should be interpreted as statistical associations rather than as evidence of temporal or causal processes.

### Moderating effects analysis

Building on the preceding path analysis, this study employed a two-stage multigroup strategy to examine whether sport type moderated the association between dependence on AI training recommendations and bodily-signal outsourcing tendency. The sample was divided into a physically dominant sport group (*n* = 196) and a skill-dominant sport group (*n* = 185). In the first stage, multigroup confirmatory factor analysis was conducted to evaluate measurement invariance across the two groups. Configural, metric, and scalar invariance were tested sequentially to determine whether the five core constructs were measured comparably across sport types. The results are presented in Table 4.

**Table 4:** Measurement invariance across sport types.

Model	*χ*^2^ (df)	CFI	RMSEA	ΔCFI	ΔRMSEA
Configural	687.873 (628)	0.989	0.016	–	–
Metric	710.840 (650)	0.989	0.016	−0.0002	−0.0001
Scalar	734.204 (672)	0.989	0.016	−0.0002	−0.0001

ΔCFI and ΔRMSEA represent changes relative to the immediately less constrained model. Measurement invariance was considered supported when |ΔCFI| ≤ 0.010 and |ΔRMSEA| ≤ 0.015.

The configural invariance model demonstrated good fit, *χ*^2^(628) = 687.873, *p* = 0.049, CFI = 0.989, TLI = 0.988, RMSEA = 0.016, 90% CI [0.001, 0.023], and SRMR = 0.048, indicating that the same five-factor measurement structure was supported across the physically dominant and skill-dominant sport groups. After the corresponding factor loadings were constrained to equality across groups, the metric invariance model also demonstrated good fit, *χ*^2^(650) = 710.840, *p* = 0.049, CFI = 0.989, TLI = 0.988, RMSEA = 0.016, and SRMR = 0.051. Relative to the configural model, the change in model fit was negligible, Δ*χ*^2^(22) = 22.967, *p* = 0.404, ΔCFI = −0.0002, and ΔRMSEA = −0.0001, supporting metric invariance. The scalar invariance model, in which item intercepts were additionally constrained to equality across groups, also demonstrated good fit, *χ*^2^(672) = 734.204, *p* = 0.048, CFI = 0.989, TLI = 0.988, RMSEA = 0.016, and SRMR = 0.051. Compared with the metric invariance model, changes in fit were again minimal, Δ*χ*^2^(22) = 23.365, *p* = 0.381, ΔCFI = −0.0002, and ΔRMSEA = −0.0001, supporting scalar invariance. Taken together, these results supported configural, metric, and scalar invariance, indicating satisfactory cross-group comparability of the five latent constructs.

After measurement invariance had been established, multigroup path analysis based on construct mean scores was conducted to compare the association between dependence on AI training recommendations and bodily-signal outsourcing tendency across sport types. Constraining this path to equality across groups resulted in a significant deterioration in model fit relative to the freely estimated model, Δ*χ*^2^(1) = 4.09, *p* = 0.043, indicating that the strength of this association differed significantly between the two groups. The standardized coefficient was higher in the physically dominant sport group (*β* = 0.59, *p* < 0.001) than in the skill-dominant sport group (*β* = 0.53, *p* < 0.001). These findings indicate that the positive association between dependence on AI training recommendations and bodily-signal outsourcing tendency was stronger among athletes in physically dominant sports, thereby supporting H6a. It should be noted that the standardized path coefficients were 0.59 and 0.53 for the two sport groups, respectively, indicating only a modest between-group difference. Accordingly, although the difference reached statistical significance, it should be interpreted with caution and regarded as preliminary evidence of variation across sport types.

This pattern was consistent with the theoretical expectations of the study. Physically dominant sports typically place greater emphasis on indicators such as training load, fatigue, recovery status, heart-rate variability, and other quantifiable performance-related data. AI-based training recommendation systems may therefore be more deeply embedded in routine monitoring and training adjustment in these sports, giving algorithmic feedback greater interpretive weight in athletes’ daily decision-making. Under conditions of sustained exposure to such data-driven feedback, athletes may become more inclined to rely on external systems when interpreting their physical condition, potentially reducing reliance on their own perceptions of fatigue, recovery, and training tolerance. By contrast, although skill-dominant sports also incorporate intelligent monitoring and feedback, training decisions often require the integration of kinesthetic information, technical rhythm, spatial awareness, and context-specific experience. Because athletes’ own interpretation of bodily and technical information remains relatively salient in these settings, the association between dependence on AI training recommendations and bodily-signal outsourcing tendency may be comparatively weaker.

## Discussion

### Association between dependence on AI training recommendations and bodily-signal outsourcing

The results showed that AI training recommendation dependence was significantly and positively associated with body signal outsourcing tendency. Athletes who reported greater reliance on AI-generated recommendations also tended to report a stronger tendency to delegate the interpretation of fatigue, pain, recovery, and movement-related states to external systems. This finding is consistent with cognitive offloading theory, which proposes that individuals may use external tools to reduce cognitive demands by transferring portions of monitoring and judgment tasks to the external environment or technological systems (Risko and Gilbert, 2016). In competitive training, athletes typically interpret bodily signals by integrating immediate sensations, accumulated training experience, training-load information, and technical performance. AI recommendation systems provide an additional source of timely, data-driven information for these judgments. When athletes consistently prioritize such recommendations, greater AI training recommendation dependence may coexist with a stronger tendency to rely on external systems when interpreting bodily states.

These findings further suggest that the psychological characteristics associated with AI training recommendation dependence extend beyond simply “trusting the system” or “using the technology” and may also involve differences in how athletes interpret bodily information. In conventional training contexts, athletes continually learn to recognize fatigue thresholds, distinguish normal discomfort from potential warning signs, and develop bodily awareness through accumulated training experience. AI systems can supplement these judgments by presenting physiological information through quantitative indicators, risk alerts, and individualized training recommendations. However, when athletes rely more heavily on system-generated information to determine whether they are fatigued, whether training should be adjusted, or whether adequate recovery has occurred, their bodily sensations may increasingly function as information to be verified against external feedback rather than as an independent basis for training-related judgment.

From a human–AI decision-making perspective, these findings also suggest that the psychological characteristics associated with AI-assisted training may be more closely related to patterns of reliance on AI-generated recommendations than to the technology itself. The appropriate reliance framework emphasizes that effective human–AI collaboration requires individuals to use AI recommendations when they are sufficiently reliable while retaining the capacity for autonomous judgment when such recommendations are uncertain, unavailable, or inconsistent with their own assessments (Schemmer et al., 2023). In the present study, higher levels of AI training recommendation dependence were associated with a stronger body signal outsourcing tendency. This pattern suggests that when AI-generated recommendations become a prominent basis for interpreting bodily states, human–AI interaction may reflect a greater degree of substitution for personal judgment rather than merely support for such judgment. Accordingly, continuous, objective, and real-time data feedback may coexist with lower levels of active integration of bodily information and a stronger tendency to rely on external systems when interpreting bodily states.

### Association between bodily-signal outsourcing and reduced self-regulatory experience

The results showed that body signal outsourcing tendency was significantly and positively associated with reduced sense of self-regulation. Athletes who relied more heavily on AI systems to interpret bodily signals such as fatigue, pain, recovery, and movement-related states also tended to report a weaker sense of active control and self-regulation during training. This finding is consistent with the conceptualization of autonomy in self-determination theory. According to self-determination theory, autonomy refers to the experience of acting in accordance with one’s own volition, self-understanding, and internal judgment (Deci and Ryan, 2000; Ryan and Deci, 2017). In training contexts, athletes’ sense of self-regulation is closely related to their ongoing interpretation of bodily states, training demands, and movement-related adjustments. When assessments of bodily states rely more heavily on external confirmation, athletes may continue to perform prescribed training tasks while experiencing a relatively weaker sense of personal control over how those tasks are evaluated and regulated.

Body signal outsourcing may therefore be associated with meaningful differences in athletes’ perceived agency during training. Fatigue, pain, and recovery are highly context dependent, and the meaning of a given physiological signal may vary across training phases, sport-specific demands, and individual experience. Athletes therefore need to develop the capacity to interpret their own bodily responses through accumulated training experience. AI systems can supplement this process by providing more explicit and quantifiable information. However, greater reliance on external systems for interpreting bodily states may coexist with a decision-making pattern in which athletes place greater weight on system-generated assessments and relatively less weight on their own interpretation of bodily information. Such a pattern may be associated with a weaker sense of personal agency in training regulation and greater reliance on external cues.

These findings further suggest that the availability of data-driven feedback does not necessarily correspond to a stronger experience of athlete autonomy. AI-generated recommendations may function as useful resources when they are used to supplement, calibrate, or extend athletes’ own judgments. By contrast, when such recommendations become the primary reference for evaluating bodily states, greater reliance on external interpretation may coexist with a more pronounced reduction in perceived self-regulation. The critical issue may therefore concern not simply the amount of information provided by AI systems, but the extent to which athletes retain interpretive authority over that information. When training-related judgments depend more heavily on system-generated prompts, lower confidence in one’s own bodily sensations may coexist with a weaker perceived capacity to independently adjust training load, communicate discomfort, and evaluate recovery status.

### Sequential associations among reduced self-regulatory experience, training decision hesitancy, and diminished training self-efficacy

The results showed that reduced sense of self-regulation was significantly and positively associated with training decision hesitation, which was, in turn, significantly and positively associated with reduced training self-efficacy. In addition, a significant serial indirect association was observed along the pathway “AI training recommendation dependence → body signal outsourcing tendency → reduced sense of self-regulation → training decision hesitation → reduced training self-efficacy.” These findings indicate a continuous pattern of statistical associations linking AI training recommendation dependence to psychological characteristics related to athletes’ autonomous judgment, encompassing bodily perception, regulatory control, decision-making, and efficacy beliefs. Higher levels of AI training recommendation dependence may coexist with a stronger tendency to seek external confirmation, which may also be associated with lower levels of initiative in training regulation and greater hesitation in specific decision-making situations.

From the perspective of the appropriate reliance framework in human–AI decision-making, effective AI-assisted decision-making does not require the indiscriminate adoption of AI recommendations. Rather, it depends on users’ ability to determine when such recommendations are sufficiently reliable and when they should be reconsidered or adjusted in light of personal experience and contextual information (Schemmer et al., 2023). In the present study, reduced sense of self-regulation was significantly and positively associated with training decision hesitation. One possible explanation is that athletes who place less confidence in their own bodily sensations and training experience may find it more difficult to balance AI-generated recommendations with their own judgment. When AI recommendations are unavailable, ambiguous, or inconsistent with bodily sensations, these athletes may report greater training decision hesitation, reflected in behaviors such as waiting for system confirmation, repeatedly checking available data, or seeking additional external reassurance rather than independently adjusting their training on the basis of bodily experience. This pattern is consistent with the theoretical emphasis of the appropriate reliance framework on maintaining an appropriate balance between external advice and autonomous judgment.

The significant positive association between training decision hesitation and reduced training self-efficacy can also be interpreted from the perspective of self-efficacy theory. Self-efficacy refers to an individual’s belief in their capability to successfully perform a task and is shaped, in part, by mastery experiences, perceived control, and confidence in managing task demands (Bandura, 1997). In training contexts, athletes who are able to adjust training load, communicate discomfort, and modify movements on the basis of their own bodily states may report stronger confidence in their ability to evaluate training conditions and make appropriate decisions. By contrast, greater reliance on external confirmation and more pronounced training decision hesitation may coexist with lower confidence in one’s ability to regulate training independently. Such differences in efficacy beliefs may become particularly salient when AI-generated recommendations are unavailable. Overall, the present findings support a consistent association between training decision hesitation and reduced training self-efficacy; however, the temporal ordering and causal direction of these relationships require further examination using longitudinal or experimental designs.

### Contextual boundary of AI training recommendation dependence across sport types

The results showed that sport type significantly moderated the association between AI training recommendation dependence and body signal outsourcing tendency. Specifically, this association was stronger in physical-dominant sports than in skill-dominant sports, suggesting that its magnitude varies across sport contexts. Such variation may be related to differences in training structure, forms of bodily feedback, and the intensity of data monitoring across sports.

This pattern can be interpreted from the perspective of cognitive offloading theory. The extent to which individuals rely on external tools is partly related to the frequency, stability, and perceived authority of those tools within a given task environment (Risko and Gilbert, 2016). Physical-dominant sports typically place greater emphasis on training load, fatigue accumulation, recovery status, and changes in physical performance. These characteristics are readily represented through quantitative indicators such as heart rate, speed, power output, lactate concentration, and sleep-related recovery metrics. In such contexts, AI systems can continuously provide relatively explicit assessments of athletes’ physical condition and corresponding recommendations for training-load adjustment. Athletes may therefore place greater weight on system-generated information when interpreting their bodily states. This interpretation is consistent with the stronger association between AI training recommendation dependence and body signal outsourcing tendency observed in physical-dominant sports.

By contrast, although skill-dominant sports also incorporate data-driven feedback, training decisions are less readily captured by isolated physiological indicators. Movement rhythm, technical feel, spatial awareness, competitive context, and situational adjustment all require substantial experiential and context-sensitive judgment. Bodily information in these sports is often closely integrated with technical execution, movement control, and changing task demands, making it more difficult for AI-generated recommendations to serve as a comprehensive substitute for athletes’ own interpretations. Accordingly, even when athletes in skill-dominant sports rely on AI-generated recommendations, such reliance may function primarily as an additional source of technical or training information and may therefore be less strongly associated with body signal outsourcing tendency.

From the perspective of the appropriate reliance framework, these between-group differences further suggest that human–AI reliance should be understood within the specific demands of each training context. The association between AI training recommendation dependence and body signal outsourcing tendency may depend not only on the perceived accuracy of the system but also on the extent to which athletes retain the capacity to evaluate the applicability of AI-generated recommendations within their sport-specific context (Schemmer et al., 2023). In physical-dominant sports, the correspondence between quantitative indicators and training-related judgments may be relatively direct, which may encourage athletes to treat system-generated recommendations as an important basis for evaluating their physical condition. In skill-dominant sports, by contrast, AI-generated feedback often requires further interpretation through technical expertise and situational judgment, potentially allowing greater scope for athletes’ own judgment to remain involved in training decisions.

Overall, these findings suggest that the association between AI training recommendation dependence and body signal outsourcing tendency is context dependent. In physical-dominant sports, where training is often more intensively quantified, AI training recommendation dependence may be more closely associated with reliance on external systems for interpreting bodily states. In skill-dominant sports, where bodily perception is more closely integrated with technical expertise and situational judgment, this association appears comparatively weaker. These findings therefore help clarify the contextual applicability of the proposed serial association model and suggest that psychological characteristics related to athletes’ autonomous judgment should be examined in relation to sport-specific training structures and patterns of technology use. It should be emphasized, however, that these between-group differences reflect variation in path coefficients observed in cross-sectional data and should not be interpreted as evidence that sport type causes differences in psychological outcomes.

## Conclusion and recommendations

### Conclusion

Drawing on cognitive offloading theory, self-determination theory, and the appropriate reliance framework in human–AI decision-making, this study developed and tested a serial association model linking AI training recommendation dependence, body signal outsourcing tendency, reduced sense of self-regulation, training decision hesitation, and reduced training self-efficacy. Based on cross-sectional questionnaire data from 381 athletes and the results of confirmatory factor analysis, path analysis using construct mean scores, and multigroup analysis, the following conclusions were drawn:

(1) AI training recommendation dependence was significantly and positively associated with body signal outsourcing tendency. Athletes who reported greater reliance on AI-generated training recommendations also tended to report a stronger tendency to rely on external systems to interpret bodily signals, including fatigue, pain, recovery, and movement-related states. This finding indicates a significant association between AI training recommendation dependence and the externalization of bodily-state interpretation.

(2) Body signal outsourcing tendency was significantly and positively associated with reduced sense of self-regulation. Athletes who relied more heavily on AI systems to interpret their bodily states also tended to report lower levels of perceived self-regulation. This finding suggests that body signal outsourcing tendency may represent an important psychological characteristic associated with reduced sense of self-regulation in intelligent training contexts.

(3) Reduced sense of self-regulation was significantly and positively associated with training decision hesitation, which was, in turn, significantly and positively associated with reduced training self-efficacy. Athletes reporting lower levels of perceived self-regulation also tended to report greater hesitation, waiting, and repeated confirmation when adjusting training, responding to physical discomfort, or encountering ambiguous AI recommendations. Greater training decision hesitation was also associated with lower training self-efficacy, indicating a continuous pattern of statistical associations among these variables.

(4) A significant serial indirect association was identified between AI training recommendation dependence and reduced training self-efficacy through body signal outsourcing tendency, reduced sense of self-regulation, and training decision hesitation. These findings reveal a continuous association pattern linking technology dependence, body signal outsourcing, reduced self-regulation, decision hesitation, and reduced self-efficacy. Given the cross-sectional design, however, this pattern should be interpreted as a statistically significant serial indirect association rather than as evidence of a temporal or causal chain.

(5) Multigroup analysis indicated a significant between-group difference in the path linking AI training recommendation dependence to body signal outsourcing tendency. This association was stronger in physical-dominant sports than in skill-dominant sports, suggesting that its magnitude may vary across sport contexts. Such variation may be related to differences in training structure, forms of bodily feedback, and the intensity of data monitoring. These findings further suggest that the associations among psychological characteristics in intelligent training contexts are not entirely homogeneous and may be bounded by sport-specific training conditions.

### Recommendations

Based on the findings of this study, the application of AI training recommendation systems in competitive sports should extend beyond improving data accuracy and optimizing training efficiency to include consideration of athletes’ bodily awareness, self-regulation, and autonomous training judgment. Given the observed pattern of associations linking AI training recommendation dependence with body signal outsourcing tendency, reduced sense of self-regulation, training decision hesitation, and reduced training self-efficacy, the following recommendations are proposed to mitigate the potential risks associated with excessive reliance on AI and diminished autonomous judgment.

(1) Establish a training decision-making process of “self-assessment first, AI review second.” Before each training session, athletes should independently complete a brief assessment of their current condition, including perceived fatigue, pain, recovery status, psychological readiness, and confidence in training. This assessment may be conducted using a brief checklist or a 1–10 rating scale. Athletes should then review the recommendations generated by the AI system and compare them with their initial self-assessment. Coaches may further encourage athletes to identify discrepancies between the two sources of information and briefly explain the basis for their own judgments. This procedure may help preserve athletes’ active involvement in interpreting their bodily states before external algorithmic information is introduced.

(2) Establish a structured system for recording and reviewing bodily signals. Training teams may adopt a three-column record comprising “Bodily Signals—AI Feedback—Actual Training Response.” The first column records athletes’ subjective bodily sensations, the second documents AI-generated feedback or recommendations, and the third records actual training performance and subsequent recovery status. Coaches, strength and conditioning specialists, or sport psychologists may conduct brief weekly review sessions to examine situations in which athletes’ own judgments corresponded more closely with subsequent outcomes and situations in which AI-generated recommendations provided greater informational value. Repeated comparison among subjective perception, algorithmic feedback, and actual training responses may provide athletes with opportunities to refine their interpretation of bodily signals while reducing uncritical reliance on external systems.

(3) Position AI recommendations as supporting evidence rather than direct instructions. Coaches should avoid presenting AI-generated outputs as definitive training decisions. Instead of simply stating that “the system recommends reducing the training load” or “the system indicates that the load can be increased,” coaches may encourage athletes to articulate their own assessments before considering algorithmic feedback. Questions such as “How do you perceive your current condition?,” “Does this recommendation correspond with your bodily sensations?,” and “How would you adjust the session if AI feedback were unavailable?” may facilitate active reflection. A three-component decision-making process integrating “athlete self-assessment—AI recommendation—coach judgment” may help ensure that AI functions as a source of decision support rather than as the sole basis for training adjustments.

(4) Incorporate selected training sessions with delayed or temporarily unavailable AI feedback. During relatively low-risk training sessions, coaching staff may designate specific periods in which athletes do not immediately access AI-generated recommendations and instead regulate training load, movement execution, or pacing on the basis of bodily sensations and accumulated training experience. AI feedback can subsequently be reviewed for comparison and calibration. Such practices should be introduced cautiously and should not be implemented during high-load sessions or in situations involving elevated injury risk. They may be more appropriate for routine conditioning, technical practice, or recovery-oriented sessions. The purpose is not to reduce the use of AI, but to preserve structured opportunities for athletes to exercise autonomous judgment and self-regulation within appropriate safety boundaries.

(5) Give particular attention to potential AI dependence in physical-dominant sports. Because the association between AI training recommendation dependence and body signal outsourcing tendency was relatively stronger in physical-dominant sports, disciplines such as track and field, swimming, rowing, and cycling may warrant closer attention to whether athletes directly equate system-generated data with their actual bodily condition. Training teams may monitor behavioral indicators of excessive reliance, such as reluctance to adjust training in the absence of AI recommendations, disregard of personally experienced pain or fatigue when these sensations conflict with system feedback, or repeated and excessive checking of training data. Such indicators may help coaches identify situations in which AI-assisted training is associated with reduced reliance on athletes’ own bodily awareness and autonomous judgment.

### Limitations and directions for future research

This study has several limitations that should be acknowledged. First, the cross-sectional design permits the identification of statistical associations among the study variables but does not establish their temporal ordering or causal direction. Future research should examine the proposed relationships using longitudinal designs, cross-lagged panel models, or experimental approaches to provide stronger evidence regarding temporal and causal processes.

Second, all core variables were assessed through athletes’ self-reports collected at a single time point. Although the diagnostic analyses did not indicate that the observed covariance structure was dominated by a single common factor, the potential influence of common-method variance cannot be completely excluded. Future studies could strengthen methodological rigor by incorporating multiple data sources, including coach evaluations, training logs, system usage records, and behavioral indicators.

Third, the study employed a non-probability sampling strategy, with participants recruited primarily from high-level collegiate sports teams, provincial and municipal professional teams, and sports training centers. Consequently, the representativeness of the sample is limited, and the generalizability of the findings to other athlete populations requires further examination. Future research should replicate the proposed model in larger and more diverse samples drawn from different competitive levels, training systems, and geographical contexts.

Fourth, several measurement instruments were specifically developed or contextually adapted for AI-assisted training environments. Although the present study provided initial evidence of their reliability, convergent validity, and discriminant validity, their psychometric stability and broader applicability require further validation. Future research should examine these measures in independent samples and across athletes from different competitive levels, sport backgrounds, and cultural contexts.

Finally, sport type was classified into physically dominant and skill-dominant categories to capture broad differences in training-task structure and feedback characteristics. Although this classification facilitated multigroup comparisons, it may oversimplify the heterogeneity that exists across individual sports. Future studies could adopt more differentiated classification approaches by incorporating sport-specific demands, training characteristics, and patterns of AI-system use to provide a more nuanced understanding of contextual variation.

## Data Availability

The datasets presented in this study can be found in online repositories. The names of the repository/repositories and accession number(s) can be found in the article/Supplementary material.

## References

[ref1] Bandura A. (1997). Self-Efficacy: The Exercise of Control. New York, NY: W. H. Freeman.

[ref2] Bompa T. O. Haff G. G. (2009). Periodization: Theory and Methodology of Training. 5th Edn. Champaign, IL: Human Kinetics.

[ref3] Deci E. L. Ryan R. M. (2000). The “what” and “why” of goal pursuits: human needs and the self-determination of behavior. Psychol. Inq. 11, 227–268. doi: 10.1207/S15327965PLI1104_01

[ref4] Fornell C. Larcker D. F. (1981). Evaluating structural equation models with unobservable variables and measurement error. J. Mark. Res. 18, 39–50. doi: 10.1177/002224378101800104

[ref5] Gerlich M. (2025). AI tools in society: impacts on cognitive offloading and the future of critical thinking. Societies 15:Article 6. doi: 10.3390/soc15010006

[ref6] Hayes A. F. (2018). Introduction to Mediation, Moderation, and Conditional process Analysis: A Regression-based Approach. 2nd Edn. New York, NY: The Guilford Press.

[ref7] Kline R. B. (2016). Principles and Practice of Structural Equation Modeling. 4th Edn. New York, NY: The Guilford Press.

[ref8] Mateus N. Abade E. Coutinho D. Gómez M.-Á. Lago-Peñas C. Sampaio J. (2025). Empowering the sports scientist with artificial intelligence in training, performance, and health management. Sensors 25:139. doi: 10.3390/s25010139 PMC11723022 39796930

[ref9] Musat C. L. Mereuta C. Nechita A. Tutunaru D. Voipan A. E. Voipan D. . (2024). Diagnostic applications of AI in sports: a comprehensive review of injury risk prediction methods. Diagnostics 14:2516. doi: 10.3390/diagnostics14222516, 39594182 PMC11592714

[ref10] Pietraszewski P. Terbalyan A. Roczniok R. Maszczyk A. Ornowski K. Manilewska D. . (2025). The role of artificial intelligence in sports analytics: a systematic review and meta-analysis of performance trends. Appl. Sci. 15:7254. doi: 10.3390/app15137254

[ref11] Podsakoff P. M. MacKenzie S. B. Lee J.-Y. Podsakoff N. P. (2003). Common method biases in behavioral research: a critical review of the literature and recommended remedies. J. Appl. Psychol. 88, 879–903. doi: 10.1037/0021-9010.88.5.879, 14516251

[ref12] Reis F. J. J. Alaiti R. K. Vallio C. S. Hespanhol L. (2024). Artificial intelligence and machine-learning approaches in sports: concepts, applications, challenges, and future perspectives. Braz. J. Phys. Ther. 28:101083. doi: 10.1016/j.bjpt.2024.101083, 38838418 PMC11215955

[ref13] Risko E. F. Gilbert S. J. (2016). Cognitive offloading. Trends Cogn. Sci. 20, 676–688. doi: 10.1016/j.tics.2016.07.002, 27542527

[ref14] Ryan R. M. Deci E. L. (2017). Self-Determination Theory: Basic Psychological Needs in Motivation, Development, and Wellness. New York, NY: The Guilford Press.

[ref15] Schemmer M. Kühl N. Benz C. Bartos A. Satzger G. (2023) Appropriate reliance on AI advice: conceptualization and the effect of explanations Proceedings of the 28th International Conference on Intelligent User Interfaces 410–422 Association for Computing Machinery

[ref16] Schoeffer J. Jakubik J. Vössing M. Kühl N. Satzger G. (2025). AI reliance and decision quality: fundamentals, interdependence, and the effects of interventions. J. Artif. Intell. Res. 82, 1261–1290. doi: 10.1613/jair.1.15873

