# Supplementary material for: Association pathways between AI training recommendation dependence and athletes’ psychological characteristics related to autonomous judgment
Source: Front Psychol. 2026 Oct 1;17:1918082. doi: 10.3389/fpsyg.2026.1918082 (PMC13668494; doi:10.3389/fpsyg.2026.1918082)
Supplement: Supplementary file 1 [file Table_1.DOCX]

**Supplementary Table S1
五个核心构念的完整问卷条目及标准化因子载荷**

说明：所有题项均采用5点Likert评分（1 = 非常不同意，5 = 非常同意）。

| **构念** | **编号** | **测量条目** | **标准化因子载荷** |
| --- | --- | --- | --- |
| AI训练建议依赖（ATRD） | ATRD1 | 在判断是否需要调整训练时，我通常会优先参考AI系统的建议。 | 0.744 |
| AI训练建议依赖（ATRD） | ATRD2 | 如果AI系统给出的建议与我自己的判断不一致，我通常更倾向于相信系统。 | 0.784 |
| AI训练建议依赖（ATRD） | ATRD3 | 在决定训练负荷或训练节奏时，我常常需要AI系统的建议作为依据。 | 0.745 |
| AI训练建议依赖（ATRD） | ATRD4 | 当AI系统没有给出明确建议时，我会觉得很难作出训练判断。 | 0.751 |
| AI训练建议依赖（ATRD） | ATRD5 | 我习惯在作出训练调整之前先等待AI系统的反馈。 | 0.770 |
| AI训练建议依赖（ATRD） | ATRD6 | 如果没有AI训练建议，我会觉得自己难以准确判断训练状态。 | 0.763 |
| 身体信号外包倾向（BSOT） | BSOT1 | 我更愿意让AI系统判断我的身体是否处于疲劳状态。 | 0.765 |
| 身体信号外包倾向（BSOT） | BSOT2 | 我常常依靠AI系统来判断自己的身体是否已经恢复。 | 0.771 |
| 身体信号外包倾向（BSOT） | BSOT3 | 当身体出现疼痛或不适时，我更依赖系统数据来判断其严重程度。 | 0.745 |
| 身体信号外包倾向（BSOT） | BSOT4 | 在判断动作状态或身体反应是否正常时，我通常更看重AI系统的分析结果。 | 0.754 |
| 身体信号外包倾向（BSOT） | BSOT5 | 相比自己的身体感受，我更相信系统对恢复状态的判断。 | 0.803 |
| 自主调节感下降（RSSR） | RSSR1 | 在训练调整上，我越来越不确定自己的判断是否可靠。 | 0.768 |
| 自主调节感下降（RSSR） | RSSR2 | 在决定是否改变训练负荷时，我常觉得自己缺乏足够的把握。 | 0.771 |
| 自主调节感下降（RSSR） | RSSR3 | 当需要根据身体状态主动调整训练时，我常觉得难以依靠自己的判断。 | 0.714 |
| 自主调节感下降（RSSR） | RSSR4 | 我在训练中的主动调节越来越需要外部系统提示。 | 0.768 |
| 自主调节感下降（RSSR） | RSSR5 | 如果没有外部系统提示，我很难主动决定如何调整训练。 | 0.759 |
| 训练决策迟疑（TDH） | TDH1 | 当AI建议不明确时，我常常不知道是否应该调整训练。 | 0.764 |
| 训练决策迟疑（TDH） | TDH2 | 当AI建议与我的身体感受不一致时，我常会反复犹豫。 | 0.764 |
| 训练决策迟疑（TDH） | TDH3 | 在需要及时调整训练负荷或动作时，我常会等待更多数据再作决定。 | 0.769 |
| 训练决策迟疑（TDH） | TDH4 | 即使身体感觉不适，我也会等待系统或数据进一步确认后再决定是否改变训练。 | 0.782 |
| 训练决策迟疑（TDH） | TDH5 | 面对需要立即作出的训练判断，我常因缺少明确的系统建议而迟迟不能决定。 | 0.804 |
| 训练自我效能下降（RTSE） | RTSE1 | 没有AI训练建议时，我对自己能否合理安排训练缺乏信心。 | 0.758 |
| 训练自我效能下降（RTSE） | RTSE2 | 我对自己独立判断训练负荷是否合适缺乏信心。 | 0.746 |
| 训练自我效能下降（RTSE） | RTSE3 | 我担心自己无法准确判断何时需要调整训练。 | 0.782 |
| 训练自我效能下降（RTSE） | RTSE4 | 在没有系统辅助时，我对自己处理训练中的身体状态变化缺乏把握。 | 0.755 |
| 训练自我效能下降（RTSE） | RTSE5 | 我觉得自己独立完成训练判断和调整的能力不够可靠。 | 0.779 |
| 训练自我效能下降（RTSE） | RTSE6 | 我越来越怀疑自己独立判断训练状态的能力。 | 0.776 |

注：N = 381。标准化因子载荷来自五因子验证性因子分析；所有题项载荷均达到显著水平（p < .001）。测量模型拟合：χ²(314) = 343.232，p = .123，CFI = 0.995，TLI = 0.994，RMSEA = 0.016，SRMR = 0.034。
